# A novel absorbable string-loop suturing device for closing a large colonic defect spanning anastomosis after endoscopic submucosal dissection

**DOI:** 10.1055/a-2719-3172

**Published:** 2025-11-05

**Authors:** Shibo Song, Xiaolong Rao, Yunlong Cai, Xinyue Guo, Long Rong

**Affiliations:** 1 26447 Endoscopy Center, Peking University First Hospital, Beijing, China

We present a novel absorbable string-loop suturing device for closing a colonic defect after endoscopic submucosal dissection (ESD). An 84-year-old female underwent ESD for a 4.0 cm × 3.0 cm lesion spanning the colonic anastomosis, leaving a large defect ( Fig. 1 ). Video 1 demonstrates the resection and suturing process. The string-loop device comprises two sheaths, a traction thread, and a closure loop with a pretied sliding knot made of absorbable thread (R216; Jinhuan, Shanghai, China) ( Fig. 2 ). During the procedure, the outer sheath was withdrawn while a metal clip grasped the closure loop through the biopsy channel and affixed it to one edge of the defect. Afterward, only the traction thread remained in the channel, with both sheaths external, allowing unobstructed passage of a second clip to fix the loop to the opposite edge. The traction thread was pulled while both sheaths advanced, and the inner sheath was used to advance the sliding knot ( Fig. 3 ), tightening it to approximate the wound edges ( Fig. 4 ). Finally, the traction thread was pulled to detach it from the closure loop, and additional clips were applied to completely close the defect ( Fig. 5 ). The 68-minute procedure used two string-loop devices and 17 clips – 47 minutes for resection and 21 for closure. The patient resumed liquids on postoperative day 3 and was discharged the next day without complications. Histology confirmed complete resection of high-grade intraepithelial neoplasia.

**Fig. 1:**
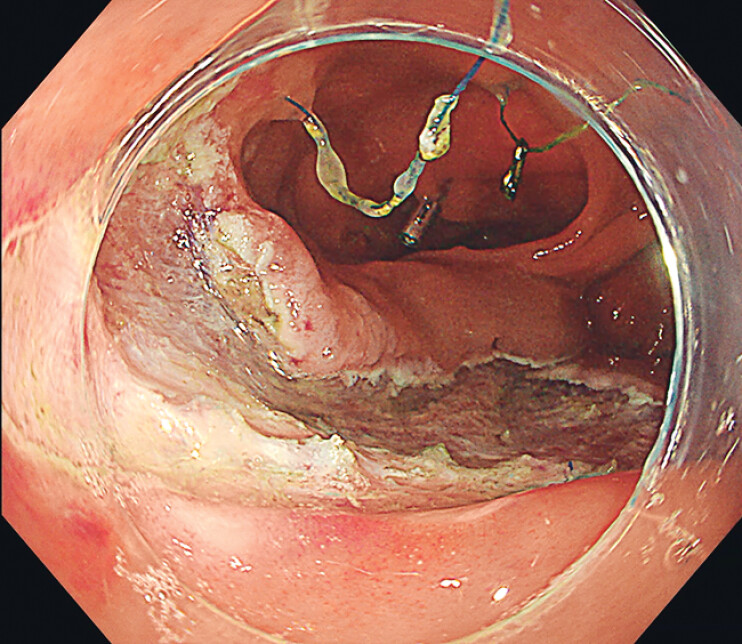
A defect spanning the anastomosis after endoscopic submucosal dissection.

A large defect spanning the anastomosis after endoscopic submucosal dissection is completely closed using a novel absorbable string-loop suturing device.Video 1

**Fig. 2:**
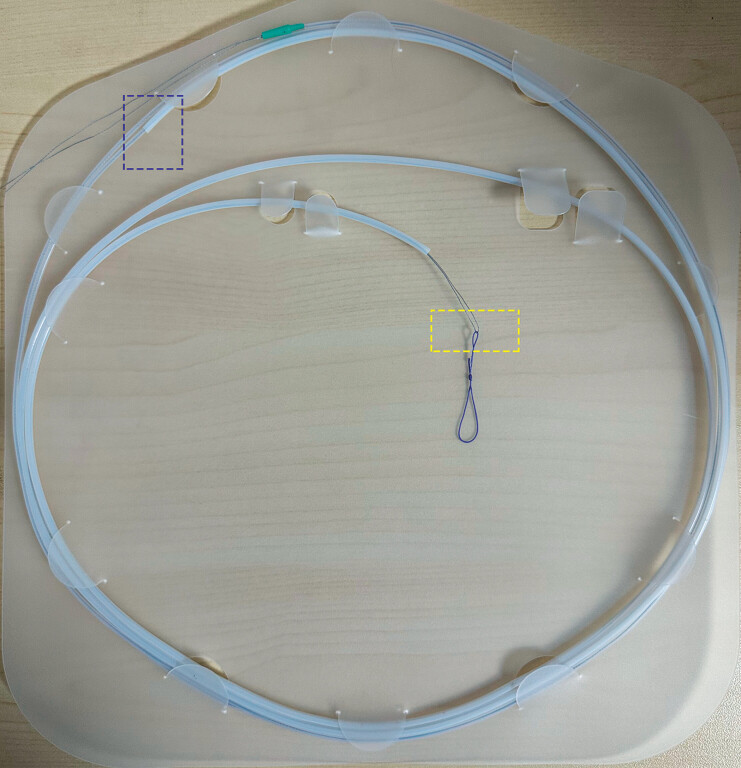
Prototype of the string-loop suturing device. Notes: The suturing device consists of three key components: 1) a purple absorbable closure loop with a pre-tied sliding knot, 2) a gray traction thread, and 3) two transparent nested polytetrafluoroethylene sheaths. Critical structural relationships are highlighted with color-coded dashed boxes: yellow marks the junction between the closure loop and traction thread, while blue indicates the overlap region of the sheaths.

**Fig. 3:**
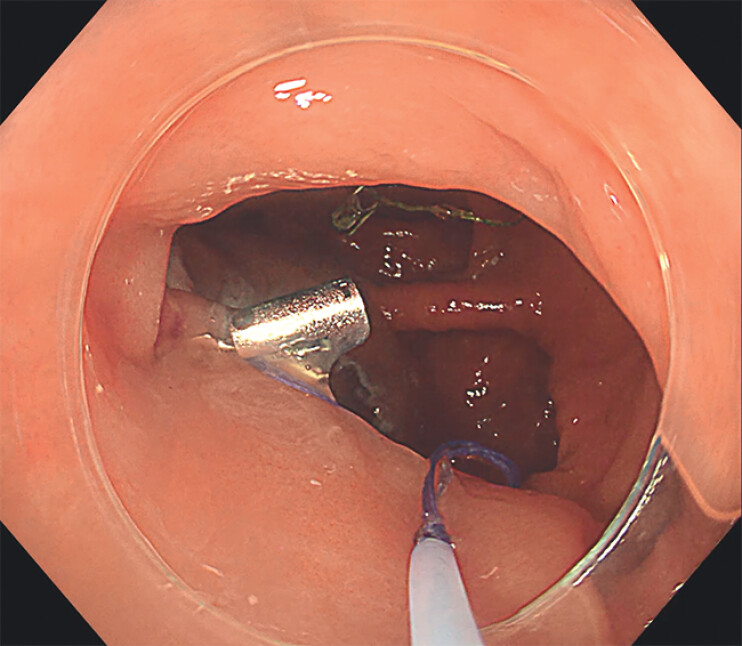
The sliding knot being advanced by the inner sheath.

**Fig. 4:**
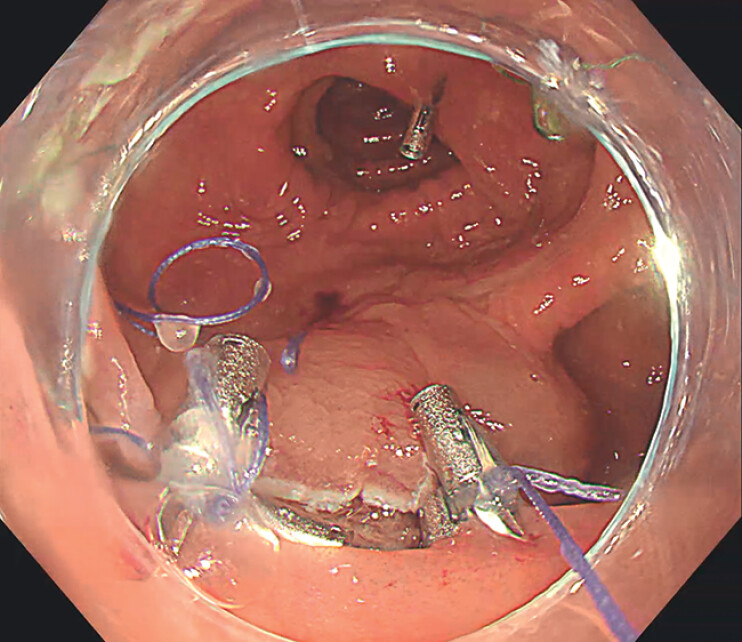
The defect was drawn together into a linear form by two string-loop suturing devices.

**Fig. 5:**
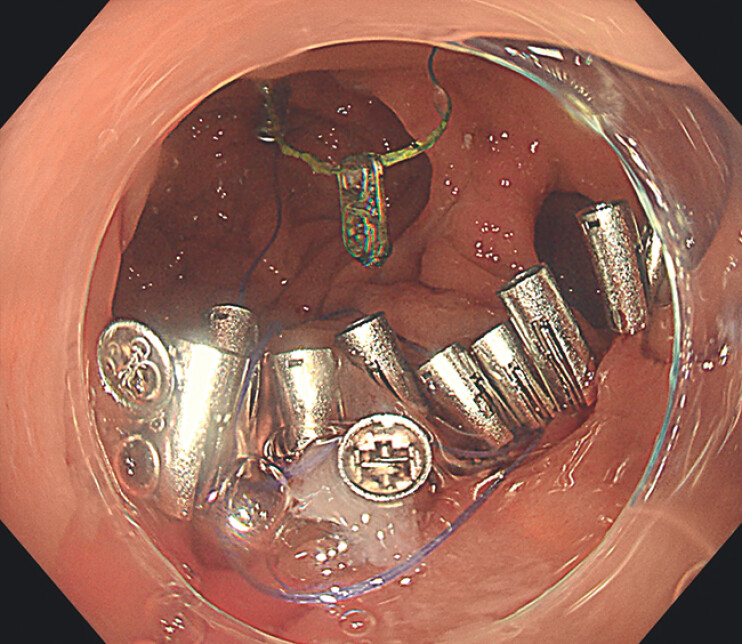
The defect completely closed with the assistance of additional clips.

Unlike nylon loop devices, which are not designed for defect closure, the string-loop device is smaller, cost-effective, and specifically designed for this purpose. The traction thread can be removed directly without scissors after tightening. Before removal, it can provide external traction without obstructing the endoscopic channel. The soft, absorbable string-loop facilitates easy clip fixation and naturally degrades within 2–3 months, promoting the clips’ detachment as the defect heals.

Endoscopy_UCTN_Code_TTT_1AQ_2AK

## Notes

Endoscopy E-Videos https://eref.thieme.de/e-videos
*E-Videos* is an open access online section of the journal *Endoscopy* , reporting on interesting cases and new techniques in gastroenterological endoscopy. All papers include a high-quality video and are published with a Creative Commons CC-BY license. Endoscopy E-Videos qualify for HINARI discounts and waivers and eligibility is automatically checked during the submission process. We grant 100% waivers to articles whose corresponding authors are based in Group A countries and 50% waivers to those who are based in Group B countries as classified by Research4Life (see: https://www.research4life.org/access/eligibility/ ). This section has its own submission website at https://mc.manuscriptcentral.com/e-videos .

